# African Perceptions of Female Attractiveness

**DOI:** 10.1371/journal.pone.0048116

**Published:** 2012-10-29

**Authors:** Vinet Coetzee, Stella J. Faerber, Jaco M. Greeff, Carmen E. Lefevre, Daniel E. Re, David I. Perrett

**Affiliations:** 1 Department of Genetics, University of Pretoria, Pretoria, South Africa; 2 Department of General Psychology and Methodology, University of Bamberg, Bamberg, Germany; 3 School of Psychology, University of St Andrews, St Andrews, United Kingdom; Tel Aviv University, Israel

## Abstract

Little is known about mate choice preferences outside Western, educated, industrialised, rich and democratic societies, even though these Western populations may be particularly unrepresentative of human populations. To our knowledge, this is the first study to test which facial cues contribute to African perceptions of African female attractiveness and also the first study to test the combined role of facial adiposity, skin colour (lightness, yellowness and redness), skin homogeneity and youthfulness in the facial attractiveness preferences of any population. Results show that youthfulness, skin colour, skin homogeneity and facial adiposity significantly and independently predict attractiveness in female African faces. Younger, thinner women with a lighter, yellower skin colour and a more homogenous skin tone are considered more attractive. These findings provide a more global perspective on human mate choice and point to a universal role for these four facial cues in female facial attractiveness.

## Introduction

The majority of studies on human behaviour focus exclusively on populations in Western, educated, industrialised, rich and democratic (WEIRD) societies [1], [2]. Results from these studies are extrapolated to explain *human behaviour* in general, despite the fact that WEIRD populations may be particularly unrepresentative of the human species as a whole [1], [2]. In order to achieve a more global perspective on human behaviour, we need to expand the literature to include other populations. Little is known about the mate choice preferences of African populations, a shortcoming this study aims to partly address.

Facial attractiveness plays a crucial role in human mating success [3] and explains more variance in overall attractiveness than bodily attractiveness [4]. Past research in WEIRD populations identified various facial cues related to female facial attractiveness: symmetry, averageness, femininity [5], youthfulness [6], skin condition [7], [8] and facial adiposity (or “facial fatness”) [9]. Despite a plethora of studies on the role of these facial cues in attractiveness in WEIRD populations (e.g. [5]–[9]), no previous study has (to our knowledge) tested the role of any of these facial features in African perceptions of African female attractiveness. Here we focus on the role of facial adiposity, skin colour, skin homogeneity and youthfulness in apparent attractiveness of African female faces in a native African population.

### 1.1 Facial Adiposity

Facial adiposity plays an important role in attractiveness judgements of British populations [9]. The relationship is curvilinear, in that overweight and underweight individuals of both sexes are judged less attractive than their normal weight counterparts [9] (see [10] for single sex analyses). Facial adiposity serves as a robust cue to health, since it is significantly related to both health judgements *and* actual measures of health (e.g. respiratory infections, antibiotics use and cardiovascular health [9]). Further studies also found a significant association between facial adiposity and health measures, such as poor general condition [11] and heart disease mortality [12]. Facial adiposity might also serve as a cue to fertility, since overweight and underweight women are less likely to conceive compared to normal weight women [13] and facial adiposity is negatively associated with salivary progesterone levels [11].

### 1.2. Skin Colour

Pale skinned women are considered more attractive than darker skinned women in a wide range of cultures [14], [15], including the African-American population [16]. Despite this, some studies find that skin tanning is considered attractive in European and American societies [17], presumably because it serves as a status symbol [18]. A lighter skin colour might serve as an indicator of fertility, as skin darkens with age, as well as in the luteal phase of the menstrual cycle and during pregnancy [14], [19]. In addition, skin lightness might serve as a cue to femininity and health, since women generally have a lighter skin colour than men [14], and European [20] and African [21] observers increase skin lightness to increase apparent health in same ethnicity faces.

Recent findings indicate that skin measuring higher on the CIELab b* colour axis, which indicates a yellower skin tone, increases men’s facial attractiveness in African and European populations [22]. Enhanced yellowness in human skin has primarily been attributed to an increase in carotenoids [21]. These are yellow and red skin pigments obtained from fruit and vegetables that are deposited in the skin [23]. Yellowness might serve as a cue to health since European [20], [21] and African [21] observers increase skin yellowness to increase apparent health in same ethnicity faces, and plasma carotenoids levels decrease in individuals with HIV and malaria [24].

European and African participants increase skin colour along the CIELab a* colour axis (which produces a slightly redder skin tone) to make European and African faces appear healthier [25]. A slightly redder skin tone serves as a cue to increased skin blood perfusion and oxygenation [25]. Since judgements of attractiveness and health are closely correlated (e.g. [26]) one might expect that increased redness might also be considered more attractive. Yet, the relationship between redness and attractiveness judgements is unclear. Re *et al.* [27] indicated a close association between the preference for oxygenated blood colouration in health and attractiveness judgements. On the other hand, two other studies did not find a significant association between skin redness and attractiveness in female European [28] or male African and European faces [22].

### 1.3 Skin Homogeneity

Homogenous (smooth) skin–particularly a homogenous skin colour distribution–positively contributes to European attractiveness judgements of shape standardised female faces [28], [29] and cropped female skin images [30], but not unmanipulated female faces [28]. Skin colour homogeneity serves as a cue to health, youthfulness and cumulative UV damage [29]–[31] and might depict healthy hormonal levels [28]; elevated levels of serum testosterone, progesterone, glucocorticoids, insulin and decreased levels of estrogen are associated with acne vulgaris, the most common form of acne [32].

### 1.4 Youthfulness

Men generally prefer to marry younger women [33] and judge younger looking female faces as significantly more fertile and attractive [6], [34]. Youthfulness serves as a valuable cue to fecundity in sexually mature women because women have a relatively small ‘reproductive window’ compared to men who stay fertile throughout most of their adult lifespan [35].

### 2. South African Environment

Attractiveness preferences for female faces can change facultatively in response to a variety of factors [36], [37], including environmental and conditional factors such as health, fertility [5], [38] and resource availability [39]. South Africa is a country that differs vastly from Western developed countries where preference studies are normally conducted. First, South Africa has a much higher disease burden than Western developed countries. The average life expectancy at birth in South Africa is 54, compared to 80 in the UK [40]. Communicable diseases, especially HIV/AIDS, account for a large part of the disease burden in South Africa, with South Africans losing an average 79% of their total years of life to communicable diseases (UK 8%; [40]). South African women also have higher prevalence of obesity (42.8%), compared to women in the UK (25.2%; [40]). South Africans are expected to pay particular attention to health cues in potential sexual partners because of a high HIV infection rate (17.8%; UK 0.2%) and lower contraceptive use (60%; UK 84%) among reproductively active adults [40].

Second, women’s total fertility rates are surprisingly similar between South Africa (2.5) and the UK (1.9), given that neighbouring African countries have much higher total fertility rates (e.g. Mozambique = 5.0; [39]). Third, the gross national income per capita, a proxy for individual resource availability, is much lower in South Africa ($ 9,790) than in the UK ($ 36,240) [40]. Finally, heavy women are considered attractive in traditional African subsistence-based societies [41], but the recent political and economic transition in South Africa might have changed attractiveness preferences amongst the young African elite. Modern African female fashion models in South Africa are significantly thinner than their white counterparts [42] and African University students report significantly more eating disorder pathology than white students in South Africa [43]. These studies might indicate a shift to a new African body ideal closely aligned to Western ideals. A lighter, yellower and redder skin colour is also considered more attractive in traditional African society even before colonial occupation [44]. In modern African society a preference for lighter skin colour is still prevalent, with young upwardly mobile African women driving the market for skin lighteners [45].

The aim of this study is to test the combined role of facial adiposity, skin colour, skin homogeneity and youthfulness–four facial features previously found to affect attractiveness in WEIRD populations– in African attractiveness judgements of unmanipulated African female faces. To our knowledge, this is the first study to test the relationship between these facial features and female attractiveness in a native African population. In addition, most studies test the relationship between individual facial cues and attractiveness in isolation (for review see [5]; but see [15] for notable exception), despite the fact that observers have access to multiple facial cues simultaneously. By including all four facial cues in a single analysis we can also assess whether the cues make independent contributions to attractiveness, or whether some correlated cues contribute similar information for attractiveness judgements.

## Materials and Methods

### Ethics Statement

This study was approved in writing by ethics committees at the University of St Andrews (PS5199; PS5740) and the University of Pretoria (EC090304-020; EC0900803-045). All participants gave written informed consent prior to taking part in the study.

### Photography and Measurements

Forty-five female African participants (Mean age = 19.84, s.d. = 1.89), a subset of 52 female African participants who completed all aspects of the study, were recruited from the University of Pretoria. The participant group included underweight (20.5%), normal weight (47.7%), overweight (15.9%) and obese (15.9%) women according to criteria developed by Gallagher *et al.* [46]. All participants were photographed (Fujifilm Finepix S5 Pro) in a custom designed booth, with a uniform Munsell N5 background and three Verivide F20 T12/D65 daylight simulation bulbs in high frequency fixtures to reduce the effects of flicker. The booth was located in a room with no other lighting. Participants were seated a set distance from the camera, asked to look straight at the camera, maintain a neutral expression and had their hair pulled back to reveal facial features. A Gretag-Macbeth Mini ColorChecker color chart was included in each frame by mounting it on a Munsell N5 painted chest board that covered the body and shoulders of participants. Images were resized, colour corrected using in-house software, manually delineated by defining 119 feature points and aligned according to interpupillary distance in PsychoMorph [47].

Participants provided information on their sex and age. We measured each participant’s body height, weight and percentage body fat using a Tanita body composition analyser SC-330STX. In addition, we used a Konika Minolta CM2600d spectrophotometer to measure participants’ facial skin colour on three separate points (left cheek, right cheek and forehead) in CIELab colour space: CIELab L*(luminance axis), CIELab a* (green-red axis) and CIELab b* (blue-yellow). Higher values on the three axes indicate lighter, redder and yellower colours respectively. CIELab values measured directly from the images produced qualitatively similar results as spectrophotometry CIELab values measured directly from the skin (Text S1).

### Experimentation

We recruited 30 African participants (14 male: mean age = 21.00, s.d. = 2.26; 16 female: mean age = 20.28, s.d. = 1.44) from the University of Pretoria to rate the unmanipulated facial images for attractiveness and weight on seven-point Likert scales (attractiveness: 1 = very unattractive, 7 = very attractive; weight: 1 = very underweight; 4 = average weight; 7 = very overweight). Images were presented in a randomised order on CRT monitors calibrated using a DataColor Spyder3Pro. Participants were asked to indicate whether they knew the person in the photograph and ratings were excluded if they did (4.1% of ratings). Weight ratings of facial images were used as a measure of facial adiposity. The images were also rated for: post-inflammatory hyperpigmentation by 9 European participants from the University of St Andrews and the University of Bamberg (5 male, 3 female, 1 unspecified); mean age = 29.22, s.d. = 10.93) on a seven-point Likert scale (1 = very low amount; 7 = very high amount), and; skin heterogeneity by 16 European participants from St Andrews (8 male, 8 female; mean age = 24.94, s.d. = 5.25) on a seven point Likert scale (1 = very homogenous, 7 = very inhomogeneous). Participants received training to identify post-inflammatory hyperpigmentation before rating the images (Text S2). Skin heterogeneity and post-inflammatory hyperpigmentation images were previewed before rating, to familiarise participants with the range and variability of images and presented in a randomised order.

### Statistical Methods

CIELab L*, a* and b* values were consistent across facial regions (all Cronbach α >0.88). We therefore averaged scores for each CIELab dimension across the three facial regions, producing a single L*, a* and b* score for each image. Inter-rater reliability was high for judgements of facial attractiveness, facial adiposity, post-inflammatory hyperpigmentation and skin heterogeneity (all Cronbach α ≥0.91). Prior to analysis, all variables were examined for accuracy of data entry, missing values, outliers, normality of their distributions and pairwise linearity [48]. We tested whether any of the variables were significantly correlated using Pearson’s correlations (two-tailed); and determined which of the independent variables predict attractiveness by fitting a simultaneous General Linear Model (GLM). We included a second order equation for facial adiposity (facial adiposity^2^) since previous studies found a curvilinear relationship between facial adiposity and attractiveness [9]. All statistical analyses were performed in SPSS 20.

## Results

All variables were normally distributed (two-tailed critical z score = ±3.29, p = 0.001), except for CIELab a* (skewness z score = –5.62; kurtosis z score = 9.69; [47]). Transformation did not successfully normalise CIELab a*, but the removal of one outlier did (skewness z = –2.42; kurtosis z = 3.18), leaving 44 cases for analysis. None of the other variables had univariate outliers at p<0.001 (two-tailed critical z score = ±3.29; [48]). All the independent variables, including facial adiposity, appear to be linearly related to attractiveness. Facial adiposity was significantly correlated with percentage body fat (r = 0.78, p≤0.0005) and body mass index (BMI; r = 0.75, p≤0.0005), indicating that observers were rating facial adiposity appropriately.

The three skin colour dimensions (CIELab L*, a* and b*) were significantly and positively inter-correlated (particularly L* and b* values). Only CIELab L* and CIELab b* were significantly correlated with facial attractiveness (Table 1). CIELab b* values were more strongly correlated with facial attractiveness than CIELab L*, although not significantly more at alpha level 0.05 (two-tailed z critical = ±1.96; Steiger’s z = –0.699; [49]). The post-inflammatory hyperpigmentation and skin heterogeneity ratings were also highly inter-correlated (Table 1). We therefore included the three colour variables and the skin post-inflammatory hyperpigmentation/heterogeneity ratings in separate PCAs. The skin colour PCA produced one colour component with eigenvalue >1, which explained 73.43% of the variance. Higher values for the colour component indicate lighter (0.92), yellower (0.95) and redder (0.68) skin tone than lower values. The skin post-inflammatory hyperpigmentation/heterogeneity PCA produced one skin heterogeneity component with eigenvalue >1, which explained 93.51% of the variance. Higher values for the heterogeneity component indicate higher post-inflammatory hyperpigmentation (0.97) and skin heterogeneity (0.97) than lower values.

**Table 1:** Pearson’s correlations.

	Attractiveness	Adiposity	CIELab L*	CIELab a*	CIELab b*	PIH	Heterogeniety
**Adiposity**	−.216	–					
CIELab L*	.363*	.095	–				
CIELab a*	.194	−.025	.383*	–			
CIELab b*	.410**	.084	.890***	.484***	–		
**PIH**	−.366*	.062	−.121	−.180	−.250	–	
**Heterogeneity**	−.421**	.139	−.124	−.162	−.251	.870***	–
**Age**	−.287†	−.137	−.017	.105	−.074	−.124	.015

† p≤0.1, * p≤0.05, ** p≤0.01, *** p≤0.001. N = 44. CIELab values indicate lightness (L*), redness (a*) and yellowness (b*), PIH indicates post-inflammatory hyperpigmentation ratings and Heterogeneity indicates Skin heterogeneity ratings.

**Figure 1:**
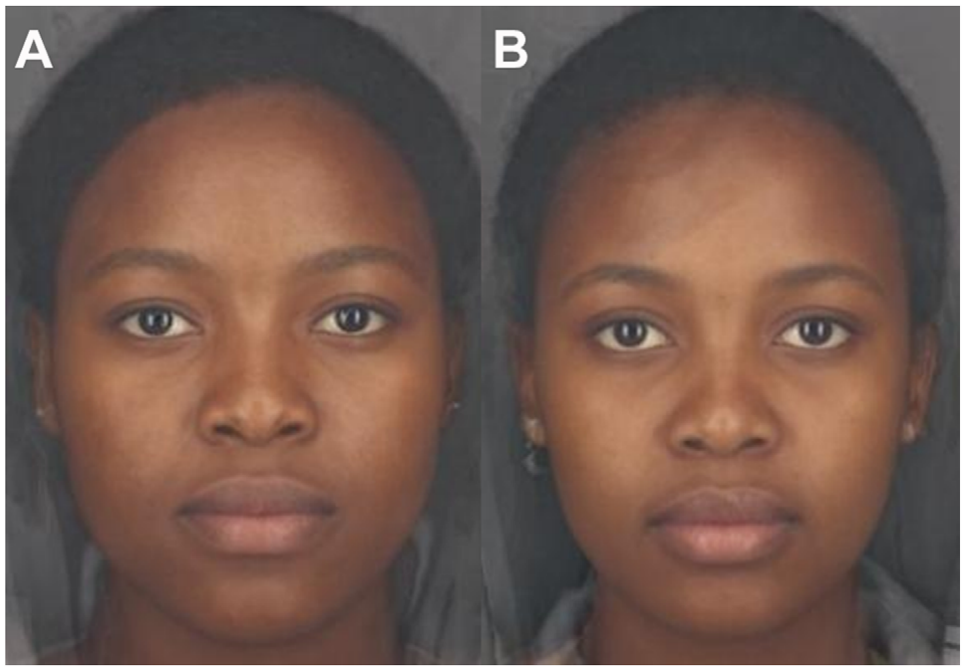
Composite images of female faces with low and high attractiveness. Composite images of the 10 women rated (A) least attractive, and (B) most attractive by African university students. Images produced with wavelet magnitude textural processing in Psychomorph. Due to the blending process involved in producing composite images, skin heterogeneity differences between the two groups are somewhat obscured.

We fitted a simultaneous GLM with attractiveness as the dependent variable and age, facial adiposity, facial adiposity^2^ and the skin colour and heterogeneity components as independent variables. No multivariate outliers were identified according to the p<0.001 criterion for Mahalanobis distance [48]. Collinearity diagnostics identified multicollinearity (Condition index = 98.90; with two variable values above 0.5) between facial adiposity and facial adiposity^2^, which was solved by centering the values as recommended by Tabachnick and Fidell [48]. The overall models significantly predicted attractiveness and explained 42% of the variance in attractiveness judgements (Table 2). Skin colour, facial adiposity, age and skin heterogeneity significantly predicted female facial attractiveness, while facial adiposity^2^ did not (Table 2). Younger, thinner women with higher values for the skin colour component (lighter, yellower and redder skin colour) and lower values for the skin heterogeneity component (more homogenous skin) were considered significantly more attractive than their counterparts (Figure 1). The predictor variables each explained roughly the same amount of variance, with age explaining the most variance, followed by skin colour, skin heterogeneity and facial adiposity (Table 1).

**Table 2:** Regression analysis of facial attractiveness judgements.

	β	F	p	Effect size
**Model**		5.510	0.001	0.420
Skin colour	0.193	6.076	0.018	0.138
Age	–0.114	7.820	0.008	0.171
Skin Heterogeneity	–0.182	4.943	0.032	0.115
Facial adiposity	–0.182	4.382	0.043	0.104
Facial adiposity^2^	0.061	0.498	0.485	0.013

Results obtained using the simultaneous regression method. Facial adiposity and facial adiposity^2^ were centered to address collinearity between the variables. Effect size: R^2^ (model); Partial eta squared (η_p_^2^; variables). N = 44.

## Discussion

Our aim was to test the combined role of four facial cues –facial adiposity, skin colour, skin homogeneity and age – as predictors of female facial attractiveness in an African population. To our knowledge, this is the first study to test which facial cues, or a combination thereof, predict attractiveness in female African faces. Our results show that when considered together age, skin colour, skin homogeneity and facial adiposity, significantly and independently contribute to what Africans consider attractive in African female faces. The fact that these four facial cues play important roles in female attractiveness preferences in European and African populations point to a more universal role for these facial cues in female attractiveness preferences.

We show that skin lightness (CIELab L*), yellowness (CIELab b*) and redness (CIELab a*) are closely correlated in African skin, but it is mainly an increased preference for a lighter, yellower skin colour that drives attractiveness preferences. A lighter, yellower skin colour may serve as a cue to health and fertility [14], [19]. Skin redness was not significantly associated with female attractiveness in African female faces; a finding that is *consistent* with previous findings in unmanipulated European female [28] and African male faces [22], but *inconsistent* with findings from studies which manipulated skin redness in facial images [20], [25], [27]. It follows that enhanced skin redness might play some role in perceptions of health and attractiveness, but its relative contribution may be overshadowed by other colour cues (i.e. yellowness and lightness).

We show that increased skin homogeneity significantly contributes to attractiveness judgements of African female faces. Previous work in European populations found a significant relationship between skin homogeneity and attractiveness in shape standardised faces and skin patches [28]–[30], but not in unmanipulated faces [28], such as the faces presented in this study.

Results show that facial adiposity significantly predicts African perceptions of attractiveness in African female faces. Contrary to previous findings in a British population [9], African participants judged thinner (e.g. lower facial adiposity) women more attractive than their heavier peers. Given that (a) the relationship between facial adiposity and attractiveness was linear, and (b) 20.5% of the images portrayed underweight women, this indicates a preference for underweight, compared to normal or overweight women. This preference for underweight women is somewhat surprising given that low body weight is often perceived as an indication of illness and disease, particularly HIV infection [50] in South Africa. The preference for thinner women is also *inconsistent* with traditional African values and low resource availability, but is *consistent* with modern media ideals, which portray a new African body ideal that is closely aligned to Western ideals [42]. This preference for thinner women might be limited to the African elite, who have better healthcare, living conditions, higher income and more access to Western media. A preference for heavy women might still be true amongst other subsets of the population [51]. Tovée *et al.* [41] found that Africans who moved to the UK within the previous 18 months prefer a significantly lower BMI than Africans living in a rural environment in South Africa, arguing that weight preferences can change fairly rapidly in response to different environmental and cultural conditions. It is also possible that those Africans who relocated to the UK already preferred a lower BMI while living in South Africa, since they were likely to come from wealthier urban environments.

In line with our predictions for health and fertility, youthfulness significantly predicted attractiveness in African female faces. Africans judged younger women significantly more attractive than older women, even within the small age range studied (18–24 years).

Most studies test the relationship between facial cues and attractiveness in isolation (e.g. [5]). By including all four facial cues in a single GLM we were able to determine the independent contributions of each facial cue to the overall attractiveness judgements. All four facial cues contributed independently to attractiveness, indicating that each of the four facial cues plays an important role in overall attractiveness and that people use multiple cues to judge attractiveness in female faces.

In summary, this is the first study to test female facial attractiveness preferences in an African population. Results show that youthfulness, skin colour, skin homogeneity and facial adiposity play crucial and independent roles in attractiveness preferences. These findings point to the universal and independent roles of these facial features in female attractiveness, despite facultative adjustments of the relationships under different environmental and cultural conditions.

## Supporting Information

Text S1 **Alternative GLM analysis using CIELab values measured directly from the face images.**
(DOCX)

Text S2 **Instructions for post-inflammatory hyper pigmentation ratings.**
(DOCX)
