# Supplementary material for: African Perceptions of Female Attractiveness
Source: PLoS One. 2012 Oct 29;7(10):e48116. doi: 10.1371/journal.pone.0048116 (PMC3483252; doi:10.1371/journal.pone.0048116)
Supplement: Text S1 — Alternative GLM analysis using CIELab values measured directly from the face images. (DOCX) [file pone.0048116.s001.docx]

**African perceptions of female attractiveness.**

Vinet Coetzee^*^, Stella J. Faerber, Jaco M. Greeff, Carmen E. Lefevre, Daniel E. Re, David I. Perrett

* Correspondence: [vcoetzee@tuks.co.za](mailto:vcoetzee@tuks.co.za)

**Supplementary Text S1: Alternative GLM analysis using CIELab values measured directly from the face images.**

Image analysis, an alternative method to spectrophotometry, is often used to measure CIELab values directly from images. For completeness we measured the average CIEL*a*b* values of two regions (cheek and forehead) of the facial images. The image colour PCA produced two colour components with eigenvalue > 1, which explained 80.91 % of the variance. L* (luminance) and b* (yellowness) values of the cheek and forehead patches loaded highly and positively on the first component (all>0.83), while a*(redness) values of the cheek and forehead patches loaded highly and positively on the second component (both > 0.73). A GLM substituting image colour factors for the spectrophotometry skin colour factor found that facial adiposity, age, skin heterogeneity and the skin L*b* colour factor significantly predicted female facial attractiveness, while facial adiposity^2^ and the skin a* colour component did not (Table S1) . Younger, thinner women with higher values for the L*b* skin colour component (lighter and yellower skin colour) and lower values for the skin heterogeneity component (more homogenous skin) were considered significantly more attractive than their counterparts. These results are qualitatively similar to the results reported in the main manuscript.

**Table S1. Regression analysis of facial attractiveness judgements.**

|  | **β** | **F** | **p** | **Effect size** |
| --- | --- | --- | --- | --- |
| **Model** | | 4.120 | 0.003 | 0.401 |
| Skin colour L*b* | 0.177 | 4.373 | 0.043 | 0.106 |
| Skin colour a* | -0.011 | 0.016 | 0.899 | 0.000 |
| Age | –0.131 | 9.430 | 0.004 | 0.203 |
| Skin Heterogeneity | –0.205 | 5.382 | 0.026 | 0.127 |
| Facial adiposity | –0.246 | 5.944 | 0.020 | 0.138 |
| Facial adiposity^2^ | 0.103 | 1.292 | 0.263 | 0.034 |
