# Supplementary material for: African Perceptions of Female Attractiveness
Source: PLoS One. 2012 Oct 29;7(10):e48116. doi: 10.1371/journal.pone.0048116 (PMC3483252; doi:10.1371/journal.pone.0048116)
Supplement: Text S2 — Instructions for post-inflammatory hyper pigmentation ratings. (DOCX) [file pone.0048116.s002.docx]

**African perceptions of female attractiveness.**

Vinet Coetzee^*^, Stella J. Faerber, Jaco M. Greeff, Carmen E. Lefevre, Daniel E. Re, David I. Perrett

* Correspondence: [vcoetzee@tuks.co.za](mailto:vcoetzee@tuks.co.za)

**Supplementary Text S2: Instructions for post-inflammatory hyper pigmentation ratings**

You will be asked to rate faces regarding post-inflammatory hyperpigmentation. Post-inflammatory hyperpigmentation is an acquired hypermelanosis occurring after cutaneous inflammation or injury that can arise in all skin types [1]. Please rate the kind of hyperpigmentation following inflammation due to acne. You will now see a view images showing post-inflammatory hyperpigmentation due to acne.

Stimulus example:


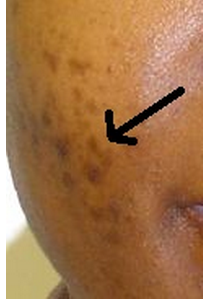


*Four stimulus examples were shown for 4 seconds each.*

**References**

1. Davis EC, Callender VD (2010) Postinflammatory hyperpigmentation: a review of the epidemiology, clinical features, and treatment options in skin of color. J Clin Aesthetic Dermatol 3: 20–31.
